# Nutritional status, food consumption, lifestyle, and physical fitness in rural and urban elementary school children in Northeast China

**DOI:** 10.3389/fnut.2022.1044877

**Published:** 2022-11-02

**Authors:** Bang Liu, Xuesheng Liu, Qi Wang, Wenjing Yan, Ming Hao

**Affiliations:** ^1^School of Public Health and Health Management, Gannan Medical University, Ganzhou, China; ^2^Liaoning Province Center for Disease Control and Prevention, Institute for the Prevention and Control of Infectious and Communicable Diseases, Shenyang, China; ^3^Key Laboratory of Prevention and Treatment of Cardiovascular and Cerebrovascular Diseases, Ministry of Education, Gannan Medical University, Ganzhou, China

**Keywords:** obesity, nutritional status, food consumption, physical fitness, China

## Abstract

Childhood obesity is observed not only in developed countries but also in some developing countries in Asia, where low physical activity and poor physical fitness have been reported. The primary goal of weight loss is to reduce body fat mass, thereby reducing the risk of metabolic syndrome. Unfortunately, a concomitant decrease in lean mass, including muscle mass, is often observed when weight is lost. This study aimed to clarify the nutritional status and physical fitness of local elementary school children and to investigate the factors associated with nutritional status. This study evaluated measures that can reduce the tendency toward obesity and recommends exercise that can reduce weight while maintaining or increasing muscle strength. A total of 911 elementary school children were recruited for this study. All the participants completed anthropometric measurements, dietary surveys, and physical fitness tests. Compared with the Chinese obesity criteria, the proportions of obese and overweight subjects were generally high [22 (rural girls) to 47% (urban boys)], and urban children had a higher obesity rate than rural children. Overall, rural children had better physical fitness test results than urban children did. Skipping rope was correlated with muscle mass. Exercise time (β = −0.31, *p* < 0.01), skipping rope (β = −0.25, *p* < 0.01), screen time (β= 0.23, *p* < 0.01); sit-ups (β = −0.20, *p* < 0.01); 400-m run (β = −0.19, *p* < 0.01); urban or rural area (β = 0.18, *p* < 0.01); oil intake (β = 0.15, *p* < 0.01), family income (β = 0.11, *p* < 0.05); and sex (β = −0.10, *p* < 0.05) were significant predictive factors for overweight and obesity, respectively. The diet of schoolchildren can be improved by reducing the intake of grain and edible oils. Physical fitness of schoolchildren can be improved by increasing exercise time and selecting exercises with higher metabolic equivalents. Rope skipping appears to be the best option because it can ameliorate obesity by increasing muscle strength. The results of this study can provide a reference for the development of obesity intervention methods for children in China and worldwide.

## Introduction

Global childhood obesity rates have considerably increased (1). From 1975 to 2016, the prevalence of obesity among children aged 5–19 increased from 0.7 to 5.6% in boys and from 0.9 to 7.6% in girls (2). In 2016, an estimated 12.4 million children were classified as obese (2). With economic development, the obesity problem in Chinese children cannot be ignored. From 1995 to 2014, the rate of overweight and obesity among Chinese children increased from 4.4 to 21.1% (3).

Poor physical fitness in children deserves further attention. According to a review of 137 studies conducted in 19 countries, cardiorespiratory fitness in children aged 9–17 decreased significantly in most countries between 1981 and 2014, as measured by the 20-m shuttle run test (4). A strong tendency toward obesity/overweight has also been observed in Asian children, and levels of physical activity and strength have been reported to be low (5–7). The 20-m shuttle-run test results of Chinese elementary school students were lower than the normative reference values that were developed based on 1,142,026 children from 50 countries (8).

Previous studies have found that poor physical fitness and obesity are important factors for high blood pressure, coronary heart disease, and stroke (9, 10). Although the symptoms usually manifest in midlife, these diseases develop progressively over time, with some signs appearing as early as childhood or adolescence (10). It is important to establish high fitness potential and sustainable lifestyle habits during childhood and adolescence. A recent prospective study showed that moderate and vigorous physical activity levels can be predicted for up to 6 years by physical fitness and fundamental movement skills developed in early adolescence (11). Additionally, it is important to establish baseline measurements of body composition and physical fitness at a young age to monitor and prevent obesity and poor physical fitness.

Exercise is an important means to ameliorate obesity. Several studies have confirmed the effectiveness of exercise in ameliorating obesity (12–14). However, many studies considered only the change in weight and ignored muscle mass (12–14). The primary goal of weight loss is to reduce body fat mass, thereby reducing the risk of metabolic syndrome (15). Unfortunately, a concomitant decrease in lean mass, including muscle mass, is often observed when weight is lost (15). Because declining muscle mass can promote poor physical fitness, weight loss that maintains or increases muscle strength is desirable for the development of an effective weight-loss and physical fitness program.

The purpose of this study was to explore the factors associated with nutritional status and provide a theoretical basis for the formulation of obesity intervention programs for primary school students.

## Materials and methods

### Participants

This cross-sectional study selected one of eight urban elementary schools and two of three rural elementary schools with the largest number of students in one district of Benxi City, Liaoning Province, in Northeast China. Two of the four classes from each grade were chosen randomly from the urban elementary schools. All the classes were chosen from the two rural elementary schools. As the population of students aged 5 and 12 years was minimal, these students were excluded from the study. A total of 911 students (262 urban boys, 250 urban girls, 214 rural boys, and 185 rural girls) aged 6–11 years were included in the study conducted from May to July 2021.

### Anthropometric measurements

Height was measured at 0.1 cm precision using a portable stadiometer (Seca 213, Germany). Weight was measured at 0.1 kg precision using a digital scale (Tanita BC-610, Japan). Upper arm circumference (AC) was measured at 0.1 cm precision a using fiberglass tape measure. Triceps skinfold thickness (TFS) was measured at 0.5 mm precision using a skinfold adipometer (PZJ-01, China) (16). All measurements were completed by nurse BL.

Body mass index (BMI; kg/m^2^) was calculated using height and weight measurements. BMI-for-age z-scores (BMI-AZ) were calculated using World Health Organization (WHO) reference data for sex and age (17). Overweight and obesity were defined using the WHO BMI classification (18).

The arm muscle area (AMA) was calculated using the AC and TFS as follows: AMA (cm^2^) = [AC (cm) – π × TSF (mm) × 10]^2^/4π. BMI-AZ was quantified using Cole's Box-Cox transformation, calculated as follows: BMI-AZ = [(BMI/M) *L* – 1]/S^*^L, where *M* is the median of the parameters, *S* is the generalized coefficient of variation, and *L* is the power in the Box-Cox transformation.

### Physical fitness

All subjects completed the Chinese Physical Fitness Test (19), which consists of the following activities: 50- and 400-m runs, sit-and-reach test, skipping rope for 1 min, and performing sit-ups for 1 min. Fitness testing was conducted in rural and urban elementary schools in May 2021. Fitness scores were calculated based on the Chinese Physical Fitness Test (19).

### Diet and nutrition

Parents' meetings, including those with children, were held at the schools so that children could complete a food frequency questionnaire with help from their parents. Daily, weekly, monthly, and yearly dietary intake was recorded using the Chinese Food Frequency Questionnaire (12). Images of food appearing in the Standard Tables of Food Composition in China were used to estimate portion size (20). Energy intake and the intake of three major nutrients and nine food groups (grains, vegetables, fruit, meat products, eggs, fish, bean products, milk, and edible oils) were calculated using standard Chinese food composition tables (20).

### Lifestyle

Using a questionnaire, students were asked to record their average daily exercise time (min/day), screen time (min/day), and sleep time (min/day) in the last week. Exercise time was defined as the sum of the time spent exercising or playing sports. Adolescent students were asked to separately report the time spent in physical activities at school (i.e., during physical education class) and sports clubs and during their free time. To estimate screen time, adolescent students were asked to separately report the time spent watching TV, playing video games, playing mobile games, and using a computer. Sleep time included sleeping overnight and during naps.

### Sociodemographic characteristics

Information regarding education level and family monthly income was collected from the parents using a questionnaire.

### Statistical analysis

An independent *t*-test was used to verify differences in the participants' mean body composition and physical fitness test results by area (urban or rural) and sex. Pearson's correlation coefficients were calculated to assess the relationship between AMA and physical fitness test scores. Multiple regression analysis was performed using BMI-AZ as the dependent variable. The 50-m run time (seconds), long-seat body anteflexion (cm), skipping rope (times/min), exercise time (min/day), screen time (min/day), sleeping time (min/day), intake values for nine food groups (g/day), three major nutrients (g/day), energy (kcal/day), education level of the father and mother, family income (yuan/month), area, and sex were used as predictor variables. Variables were selected using the stepwise increase-and-decrease method, and a threshold *p*-value of 0.20, calculated using the likelihood ratio test. JMP Pro 16.0.0 (SAS Institute Inc.) was used to perform statistical analyses, with a significance of *p* < 0.05.

## Results

### Nutritional status of local primary school students

The prevalence of underweight among children in both rural and urban areas was <10% (Table 1). Both urban and rural children had a high overweight and obesity rate: 47% in urban boys, 33% in urban girls; 36% in rural boys, and 21% in urban girls (Table 1).

**Table 1:** BMI category, 5 items of physical fitness and food consumption for urban and rural children^†^.

	**Boys**		**Girls**	
	**Urban**	**Rural**	**Urban**	**Rural**
**BMI category (kg/m** ^**2**^ **)**				
Underweight	8 (3%)	5 (2%)	16 (6%)	14 (8%)
Normal	130 (50%)	132 (62%)	152 (61%)	130 (70%)
Overweight and obese	124 (47%)	77 (36%)	82 (33%)	41 (22%)
**Results of 5 items of physical fitness test** ^‡^				
50 m run (s)	10.7 ± 1.5	10.6 ± 2.3	11.0 ± 1.5	10.7^*^± 2.1
Body anteflexion in sitting (cm)	2.3 ± 6.0	4.4^**^± 5.4	6.8 ± 5.9	7.5 ± 5.0
Skipping rope (*n*)	48.5 ± 43.6	64.5^**^± 40.7	71.0 ± 40.4	92.7^**^± 50.0
Sit ups (*n*)	25.5 ± 10.3	28.4^**^± 11.9	25.1 ± 10.5	25.6 ± 12.0
400 m run (s)	110.4 ± 19.7	105.0^*^± 16.7	112.5 ± 19.4	106.3^**^± 15.5
**Food consumption**				
Energy (kcal)	2274.2 ± 675.4	2027.4^**^± 499.8	2174.2 ± 579.6	1898.1^**^± 391.7
Proteins (g)	76.9 ± 24.9	64.7^**^± 15.8	74.0 ± 22.4	61.8^**^± 11.1
Fat (g)	87.4 ± 34.0	58.8^**^± 21.1	84.5± 31.5	54.8^**^± 20.5
Carbohydrate (g)	297.9 ± 90.0	316.7 ± 82.7	286.6 ±73.0	291.9 ± 60.8

* t-test, p < 0.05;

** t-test, p < 0.01.

† Values are n (%) or mean ± SD.

‡ All the students conducted the 50 meters run, body anteflexion in sitting and rope skipping test. The number of participants: Urban boys: 262; Urban girls: 250; Rural boys: 214; Rural girls: 185. Students in the fifth and sixth grades conducted the sit ups test. The number of participants: Urban boys: 186; Urban girls: 179; Rural boys: 150; Rural girls: 130. Students in the sixth grade conducted the 400 meters run test. The number of participants: Urban boys: 98; Urban girls: 99; Rural boys: 86; Rural girls: 70.

### Physical fitness test results

**Figure 1:**
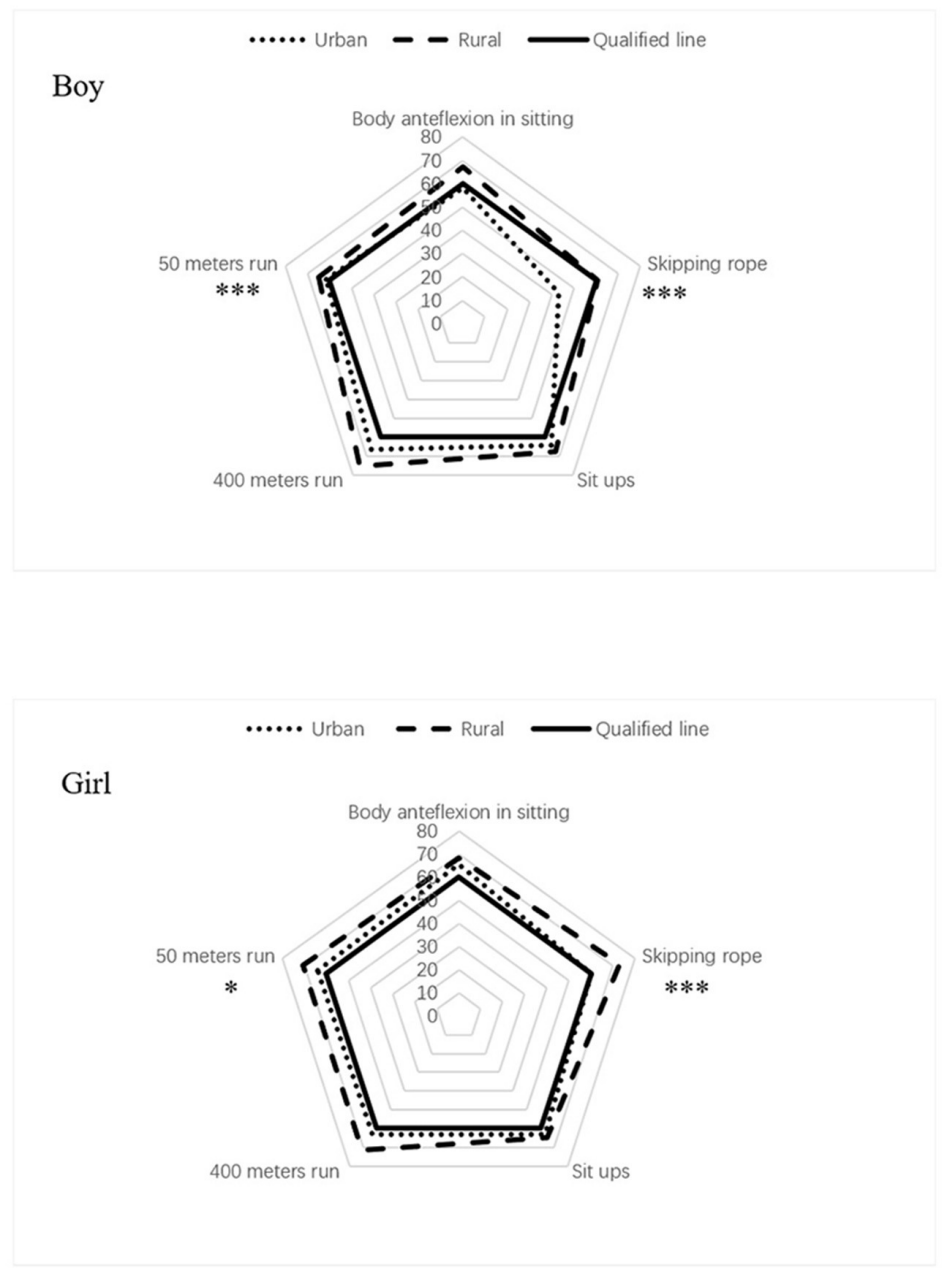
The average score of physical fitness test. *Urban vs. rural, *t*-test, and *p* < 0.05; ***Urban vs. rural, *t*-test, and *p* < 0.001.

The means and standard deviations for the physical fitness test results by area are shown in Table 1. Overall, rural children had better physical fitness test results than did urban children (Table 1). The scores for the five physical fitness test items are presented in Figure 1. Except for the skipping rope score, the mean scores exceeded the cutoff values for passing marks for urban boys. Mean scores for skipping rope and long-seat body anteflexion differed significantly between rural and urban boys, and mean scores for skipping rope and 50-m run time differed significantly between rural and urban girls. The failure rate of rural children was lower than that of urban children. In particular, the proportion of urban children with failing scores for skipping rope was 47%, which was much higher than that of other groups (Figure 1).

To clarify the relationship between muscle mass and physical fitness, we examined the correlations between AMA and individual physical fitness test scores. There was a significant positive correlation between skipping rope and AMA in all groups (*r* = 0.17–0.25, *p* < 0.05; Table 2).

**Table 2:** Correlation between results of 5 items of physical fitness test and arm muscle area (AMA).

	**Boys**		**Girls**	
	**Urban**	**Rural**	**Urban**	**Rural**
50 m run (s)	−0.03	−0.05	0.03	0.15
Body anteflexion in sitting (cm)	−0.10	0.10	−0.06	0.17^*^
Skipping rope (*n*)	0.25^**^	0.24^**^	0.17^*^	0.21^*^
Sit ups (*n*)	0.00	0.10	0.16	0.14^*^
400 m run (s)	0.04	0.24	−0.04	0.21

* Pearson's correlation coefficients, p < 0.05;

** Pearson's correlation coefficients, p < 0.01.

### Diet and nutrition of primary school students

Energy, protein, and fat intakes of urban children were significantly higher than those of rural children (Table 1). The intakes of the nine food groups were determined by area and sex (Table 3). Compared with the Chinese recommended dietary allowance (21), the intake of grains, meat, eggs, beans, and edible oils of local children was higher, whereas the intake of fruit, fish, and milk was lower. Milk intake was extremely low among rural children (15.4% in boys and 10.1% in girls; Table 3) relative to the recommended dietary allowance of 300 g. The intake of vegetables was within the recommended range. In urban children, the mean intake values for all food groups were higher than those in rural children, but significant differences were observed only for meat and milk (*p* < 0.05; Table 3).

**Table 3:** Daily intake of nine food groups (g).

**Food groups^†^**	**Recommendation^†^**	**Boys**		**Girls**	
		**Urban (*n* = 262)**	**Rural (*n* = 214)**	**Urban (*n* = 250)**	**Rural (*n* = 185)**
		**Mean ±SD**	**Mean ±SD**	**Mean ±SD**	**Mean ±SD**
Grains	250–400	593.8 ± 155.5	583.8 ± 157.3	457.3 ± 122.4	423.1 ± 110.8
Vegetables	300–500	453.2 ± 136.8	448.8 ± 102.1	387.3 ± 119.0	349.0 ± 104.7
Fruit	200–400	185.2 ± 91.4	163.8 ± 79.2	231.8 ± 98.9	214.3 ± 79.6
Meat	50–75	167.0 ± 80.5	101.0^*^± 733	115.6 ± 70.2	90.0^*^± 57.4
Eggs	25–50	69.2 ± 33.6	68.1 ± 35.5	54.3 ± 29.9	49.3 ± 27.0
Fish	50–100	35.7 ± 20.0	20.6 ± 17.5	27.8 ± 20.0	21.0 ± 18.2
Beans	30–50	67.8 ± 31.1	67.7 ± 30.5	52.8 ± 28.7	50.4 ± 23.2
Milk	300	87.0 ± 75.4	46.3^*^± 40.3	86.8 ± 53.0	32.6^*^± 20.8
Edible oils	< 30	37.0 ± 15.1	35.5 ± 15.6	36.8 ± 19.5	33.7 ± 14.1

* t-test, p < 0.05.

† [19].

### Lifestyle of primary school students

The reported means and standard deviations (min/day) for daily exercise time, screen time, and sleep time by area and sex are shown in Table 4. There was no significant difference in sleeping time between urban and rural schoolchildren, but exercise time and screen time were significantly longer in rural children than in urban children (*p* < 0.05; Table 4). The mean daily exercise time was less than the Chinese recommended exercise time of 1 h for both urban boys and girls (22). In contrast, daily exercise time was longer than the Chinese recommended daily exercise time (22) for both rural boys and girls. Screen time was longer in urban children than in rural children for both boys and girls (*p* < 0.05).

**Table 4:** Daily exercise time, screen time and sleeping time for urban and rural children.

	**Boys**		**Girls**	
	**Urban (*n* = 262)**	**Rural (*n* = 214)**	**Urban (*n* = 250)**	**Rural (*n* = 185)**
	**Mean ±SD**	**Mean ±SD**	**Mean ±SD**	**Mean ±SD**
Exercise time (min)	41.2 ± 22.1	67.0^**^± 42.1	39.9 ± 20.0	60.7^**^± 42.1
Screen time (min)	124.8^**^± 44.2	87.4 ± 33.6	121.3^**^± 42.8	85.2 ± 32.2
Sleeping time (min)	550.1 ± 42.0	545.1 ± 42.3	553.4 ± 41.3	560.7 ± 40.1

*t-test, p < 0.05;

** t-test, p < 0.01.

### Factors associated with nutritional status

Table 5 shows the results of the multiple regression analysis of the factors that contributed to BMI-AZ. Physical fitness (five items), dietary habits (three items), lifestyle (three items), education level of the father and mother, family income (yuan/month), area, and sex were included as predictor variables. Exercise time (β = −0.31, *p* < 0.01), skipping rope (β = −0.25, *p* < 0.01), screen time (β = 0.23, *p* < 0.01), sit-ups (β = −0.20, *p* < 0.01), 400-m run (β = −0.19, *p* < 0.01), area (β = 0.18, *p* < 0.01), oil intake (β = 0.15, *p* < 0.01), family income (yuan/month) (β = 0.11, *p* < 0.05), and sex (β = −0.10, *p* < 0.05) were significant predictive factors (Table 5).

**Table 5:** Factors that contributed to the tendency toward obesity^†^.

	**β**	***t***	**VIF**	***p***
Exercise time (min/days)	−0.31	−5.56	1.78	< 0.01
Skipping rope (*n*/min)	−0.25	−5.54	1.17	< 0.01
Screen time (min/days)	0.23	4.14	1.78	< 0.01
Sit ups (*n*/min)	−0.20	−4.46	1.01	< 0.01
400 m run (s)	−0.19	4.33	1.11	< 0.01
Areas (Rural: 0; Urban: 1)	0.18	2.48	3.09	< 0.01
Oil (g/days)	0.15	3.55	1.05	< 0.01
Family income (yuan/month)	0.11	2.43	1.16	< 0.05
Sex (Boy: 0; Girl: 1)	−0.10	−2.19	1.11	< 0.05
Fat (g/day)	0.09	1.79	1.42	>0.05

† R^2^: 0.43, p < 0.01; RMES: 1.19.

n: 911.

## Discussion

The results of this study showed that the overweight and obesity rates of children in northern China are relatively high. Compared to the other groups, rural girls had the lowest proportion of overweight and obesity (Table 1). However, the overweight and obesity rate of rural girls reached 22%, higher than the national average of 21% (23).

The physical fitness of children in local areas was poor (Table 1) compared to that of 89,949 children from the 32 provinces of China in 2016 in a previous study, who performed better in all of the physical fitness tests, except for the 400-m run (24). In addition, compared with the physical fitness test results in Japanese children (25), children in Northeast China had poorer results in the 50-m run and long-seat body anteflexion (Table 1). Furthermore, the present study found that rural children performed better in physical fitness tests than urban children, supporting similar surveys in China (24) and other developing countries (26, 27).

The energy intake of urban children was higher than that of rural children, which was confirmed by the fact that the intake of nine food groups of urban children was higher than that of rural children (Tables 1, 3). This may be related to the fact that the obesity rate in urban children is higher than that in rural children (Table 1). Overweight and obesity were associated with an excessive intake of grains and edible oils (Table 5). Based on the results of the multiple regression analysis, BMI-AZ increased as the intake of grains and edible oils increased (Table 5). Traditional Chinese meals contain large amounts of grains and vegetables (28) and tend to include fried dishes. A survey of Chinese adults revealed that the intake of edible oils increases with vegetable consumption, thereby promoting obesity (29). Therefore, traditional vegetable preparations are considered to be a cause of childhood obesity.

Boys and children from high-income families were more likely to be obese (Table 5). Socioeconomic status (SES) influences food selection and physical activity and has been reported as a factor in childhood obesity (30). In developed countries, low-SES groups experience higher rates of overweight and obesity in children, whereas in developing countries, higher-SES groups experience higher rates of overweight and obesity in children (30). Individuals in China with a higher SES consume more high-calorie foods such as meat (which tends to be costlier than vegetables) than individuals with a lower SES (31). Supporting previous studies, the multiple regression analysis results showed a positive correlation between household income and overweight and obesity in children (Table 5).

The one-child policy has been considered one of the causes of the increasing childhood obesity problem in China (32). This study (Table 1) and a previous study found that the obesity rate in boys is higher than that in girls in China (33). The male/female birth ratio has increased since the one-child policy commenced in 1978, and many Chinese prefer boys (32). The culture of the paternal authority system in traditional Confucianism in China allocates more domestic resources to boys than to girls (34), and this could be one reason for the higher proportion of obese boys than girls. In Chinese society, boys' obesity may also be considered a symbol of wealth, which may reduce parents' active intervention on boys' obesity (35). At the same time, boys are less likely to participate in family work (33).

Short exercise and long screen times were risk factors for obesity (Tables 2, 5). Physical activity has been demonstrated to help children control weight and reduce behaviors linked to health problems such as cardiovascular disease, diabetes, and hypertension (36); however, screened devices are used as a dominant recreational activity for all ages, especially children (37). Regardless of participation in physical activity, screen time has a negative influence on health and is associated with health-related risk factors including obesity, cardiovascular disease, and type 2 diabetes (38). Increasing exercise time and decreasing screen time are important to reduce obesity.

Exercise is effective in reducing fat and enhancing lean body mass in children, resulting in positive effects on ameliorating obesity (39). Our results showed a significant positive correlation between the skipping rope test and BMI-AZ (Table 5). The metabolic equivalent (MET) of the skipping rope was 11.7–12.5 from the different fluctuation frequencies (40), which is ~3 times that of jogging (MET: 4.5) (40). Therefore, skipping rope could produce, in less time, the same effect on metabolic rate as jogging, making it an efficient exercise for reducing obesity. Exercise-based weight loss programs (including skipping rope) have proven effective in reducing childhood obesity (41–43), and therefore, skipping rope as an exercise can be expected to reduce obesity.

Furthermore, this study indicated that skipping rope can increase muscle mass, which is expected to increase with the amount of exercise performed (Table 2). Skeletal muscle is an important site of lipid oxidation and is the most significant contributor to basal metabolism (44). Skipping rope can increase muscle mass and therefore basal metabolism, which can contribute to the prevention of obesity. Moreover, skipping rope has been shown to increase upper and lower body strength (45). A previous study in Germany demonstrated that 5 months of skipping rope training increased whole-body coordination and complex eye-hand-leg coordination, endurance, and shoulder mobility in elementary school children (46). Therefore, skipping rope can be expected to prevent obesity, increase muscle mass, improve strength, and increase athletic performance.

## Limitations

We selected only one city, Benxi, as the study area. Therefore, it is difficult to generalize the results to other cities in Liaoning Province and northeastern China. Because this was a cross-sectional study, it was not possible to determine the causal relationships between nutritional status, diet, lifestyle, exercise performance, and the factors affecting them in elementary school children.

## Conclusion

Rural children had a better nutritional status and physical fitness than urban children. However, both urban and rural children showed high rates of obesity and poor physical fitness. Diet can be improved by reducing the intake of grains and edible oils. Physical fitness can be improved by increasing exercise time and selecting exercises with higher metabolic equivalents. Rope skipping appears to be the best option because it can ameliorate obesity by increasing muscle strength.

## Data availability statement

The original contributions presented in the study are included in the article/supplementary material, further inquiries can be directed to the corresponding authors.

## Ethics statement

The studies involving human participants were reviewed and approved by Ethical approval for the study was granted by the Gannan Medical University, China. The patients/participants provided their written informed consent to participate in this study.

## Author contributions

BL and XL: data collection, data analysis, and manuscript writing. QW: study design and data analysis. WY: study design, data collection, data analysis, and manuscript writing. MH: study design, data collection, data analysis, manuscript writing, and funding acquisition. All authors contributed to the article and approved the submitted version.

## Funding

This study was supported by the Starting Research Fund of Gannan Medical University.

## Conflict of interest

The authors declare that the research was conducted in the absence of any commercial or financial relationships that could be construed as potential conflicts of interest.

## Publisher's note

All claims expressed in this article are solely those of the authors and do not necessarily represent those of their affiliated organizations, or those of the publisher, the editors and the reviewers. Any product that may be evaluated in this article, or claim that may be made by its manufacturer, is not guaranteed or endorsed by the publisher.
